# Knowledge of Dental Students from Croatia, Slovenia, and Bosnia and Herzegovina about Dental Care of Oncology Patients

**DOI:** 10.3390/dj9110132

**Published:** 2021-11-15

**Authors:** Iva Pedic, Livia Cigic, Danijela Kalibovic Govorko, Katarina Vodanovic, Ruzica Bandic, Robert Glavinic, Ivana Medvedec Mikic

**Affiliations:** 1 Private Dental Clinic Ceprnja, Bastijanova 2a, 10000 Zagreb, Croatia; iva.pedic@gmail.com; 2 Department of Oral Medicine and Periodontology, Study Programme of Dental Medicine, School of Medicine, University of Split, Soltanska 2, 21000 Split, Croatia; 3 Clinic of Dental Medicine, University Hospital of Split, 21000 Split, Croatia; dkalibovic@gmail.com (D.K.G.); imedvedecmikic@gmail.com (I.M.M.); 4 Department of Orthodontics, Study Programme of Dental Medicine, School of Medicine, University of Split, Soltanska 2, 21000 Split, Croatia; 5 Study Programme of Dental Medicine, School of Medicine, University of Split, Soltanska 2, 21000 Split, Croatia; katarina.vodanovic@mefst.hr (K.V.); ruzica.bandic@mefst.hr (R.B.); 6 Clinic of Infectious Diseases, University Hospital of Split, Spinciceva 1, 21000 Split, Croatia; robert.glavinic@yahoo.com; 7 Department of Endodontics and Restorative Dental Medicine, Study Programme of Dental Medicine, School of Medicine, University of Split, Soltanska 2, 21000 Split, Croatia

**Keywords:** dental students, dental care, oncology, chemotherapy, radiotherapy, osteoradionecrosis

## Abstract

The central role of the dentist in the treatment of oncology patients is to care for the patient’s oral cavity before, during, and after radio/chemotherapy. The aim of this research was to determine the knowledge of dental students from five universities in three neighboring countries, Croatia (Split, Rijeka, and Zagreb), Bosnia and Herzegovina (Sarajevo), and Slovenia (Ljubljana), about oncology patients’ dental care. A total of 140 students in their fourth, fifth, and sixth year of dental medicine studies participated in this research. A questionnaire with 36 specific questions was designed for this research and included questions about dental care of oncologic patients before, during, and after the oncology therapy. Most students are familiar with the incidence and most common type of head and neck tumors, while knowledge about tumor treatment and the side-effects of radiation therapy and/or chemotherapy is weak. Students did not show satisfactory knowledge about osteoradionecrosis, which is the most serious side-effect of radiotherapy; therefore, the emphasis on additional education should be greatest in this area. Teaching staff should be aware of lack of student knowledge and try to offer more information and practice in providing dental care for oncology patients.

## 1. Introduction

The central role of the dentist in the treatment of patients with head and neck cancer is to care for the patient’s oral cavity before, during, and after radio/chemotherapy. Since the oncology patients have a treatment plan that consists of different doses of radio- or chemotherapy, a detailed dental assessment is needed, and the ideal time is before oncology therapy even starts. A dental assessment includes intra/extraoral clinical examination of the head and neck, radiographic imaging analysis, laboratory reports, and insight into the complete medical history of the patient including the medications that the patient was or is still using. In addition, all teeth with caries, periapical pathoses, and a damaged periodontium or broken and half-impacted teeth may need to be treated. The treatment includes removing caries, endodontic or periodontal therapy, and, if needed, extraction. The removal of retained teeth should also be considered, especially in younger patients where some complications can be expected with unerupted third molars. In the elderly population, above 60 years, retained teeth, if they are without symptoms or clinical signs, should be left in the bone because there are more post-extraction complications, as published in the study of Trybek et al. [1]. The best time for dental treatment is at least 3 weeks before the beginning of oncology therapy. In case of a period shorter than 10 days, and, if the patient is not in acute inflammation, teeth extractions are performed during the so-called “golden window period” after radiation [2]. Dental care before oncology therapy includes oral hygiene instructions, descaling, promoting a noncariogenic diet, prophylactic treatment with fluorine preparations, and removing all sources of irritation and infection [3].

During chemotherapy and radiation therapy, good oral hygiene is crucial for patients. All elective dental treatments should be delayed until the end of oncology therapy. However, in the case of an active dental infection, a dentist and an oncologist should make a decision and a plan for helping the patient [4]. Patients on chemotherapy without radiation therapy may receive dental care, routine or necessary, but only if their counts are stable (leukocytes at least 2000 cells/mm^3^, neutrophils >1000 cells/mm^3^, platelets >50,000 cells/mm^3^). Therefore, it is necessary to do a complete blood count (CBC) and differential blood count (DBC) on the day of dental treatment [4].

The period after oncology treatment requires frequent dental check-ups so that dentists can monitor a patient’s condition through clinical examinations and detect possible local recurrences, metastatic lesions in the head and neck area, or secondary malignant lesions. The interval of “recall” visits is estimated individually, according to the patient’s risk, but is likely to be no less frequent than 3 months, at least in the first instance [5]. Regular check-ups are performed for prevention, early diagnosis, and treatment of late radiation complications. After radiotherapy, tooth extractions and other oral surgical procedures are avoided, and the most important goal is to establish adequate fluoridation of teeth. In case that tooth extraction or some other oral surgical procedure is needed, Trybek et al. [6] suggest the technique of applying platelet-rich fibrin to the post-extraction alveolus in order to reduce the severity of post-extraction complications.

The aim of this research was to determine the knowledge of dental students from five universities in three neighboring countries, Croatia (Split, Rijeka, and Zagreb), Bosnia and Herzegovina (Sarajevo), and Slovenia (Ljubljana), about oncology patients’ dental care. Moreover, the need for additional education on this topic during the time of their study was assessed.

## 2. Material and Methods

A total of 140 subjects, 21 to 27 years old, participated in this research. They were all students of fourth, fifth, and sixth year of dental medicine studies at five different universities (Split, Zagreb, Rijeka, Sarajevo, and Ljubljana) from three countries. The participation of all respondents was voluntary. The research was approved by the Ethics Committee of the University of Split, School of Medicine.

The research consisted of 36 specific questions as an online Google form from January to April 2020. The questionnaire was specially designed for this research and included questions about dental care of oncologic patients before, during, and after the oncology therapy. Using the correct statement from the literature, question sentences were formed, and answers “I agree with the statement”, “I am not sure”, “I do not agree with the statement” were offered. Moreover, data about gender, age, and the year of study were collected. After gathering all completed questionnaires, a statistical analysis of acquired data was conducted.

For statistical data processing, the STATISTICA 11.0 software package was used. Frequency tables were calculated for each question. Kruskal–Wallis ANOVA test was used to confirm the potential difference in responses between subjects from five different faculties of dental medicine studies. A general regression model was used to determine the potential influence of predictor variables (gender, year of study, and faculty). Both correlation coefficients and their significance express the potential interdependence of dependent and predictor variables. Statistical significance in all used methods was reduced to *p* < 0.05.

## 3. Results

The mean values of the age of respondents for the five faculties were very similar. The youngest students were from Split (23.0 ± 1.0 years), and the oldest were from Sarajevo (24.4 ± 0.8 years).

As for the gender of participants in this study, 115 (82.1%) were women and 25 (17.9%) were men (Table 1). The final year students (sixth year) were most numerous (Table 2).

Dental students already learn about the dental care of oncologic patients. To the question “Did you learn about providing dental care to oncology patients as part of your studies?”, 93.8% respondents from Rijeka responded affirmatively in contrast to 50% of students from Sarajevo.

Although most respondents received some education about oncology patients, their certainty that they can provide adequate care to cancer patients was generally low. Thus, the answer to the question “Do you think you have enough knowledge to help oncology patients?” was “Yes” from only 3.3% of students from Sarajevo and 34.2% of students from Zagreb.

As for the dental medicine students’ knowledge about the most frequent carcinoma in the head and neck area, students from Zagreb showed the worst results. A total of 13.2% answered incorrectly. Students from Split answered correctly in 87.5% of cases.

To the statement, “Pre-therapy assessment of an oncology patient must include a detailed clinical examination of the oral cavity and radiological imaging.”, the largest percentage of respondents from Sarajevo answered “I agree” (96.7%), and the largest percentage of respondents from Ljubljana answered “I disagree” (12.5%).

Responses to the statement “Total radiation dose greater than 40 Gray (Gy) poses a risk to osteoradionecrosis (ORN).” showed that students do not have the required level of knowledge about the strength of radiation dose that poses a risk of ORN. Approximately half of the respondents from all faculties, except Ljubljana, answered “I am not sure” (Table 3).

To the question “Do you think that antibiotic prophylaxis, before tooth extraction after oncology therapy, is a safe protection of the patient from ORN?”, most negative answers were offered by respondents from Split (68.8%), and most positive answers were from the students from Sarajevo (60%). A large number of students from all faculties answered “I do not know”, which shows us that students do not have the required level of knowledge in this area.

To the statement “In case an emergency invasive dental procedure must be performed, and the platelet count is less than 50,000 cells/mm^3^, a platelet transfusion is required.”, most respondents answered “I agree”. In general, there is a large discrepancy in responses both within and between faculties (Table 4).

Students also showed uncertainty in the case of responses to the statement “During chemotherapy, if the granulocyte count is less than 2000 cells/mm^3^, antibiotic prophylaxis is required.” (Table 5). However, it can be seen that more than half of the students, except those from Ljubljana, agreed that antibiotic prophylaxis is needed.

In terms of responses to the statement “The ideal time for extraction of a tooth with a poor prognosis is 3 weeks before the start of oncology treatment.”, there was a wide range of responses. The highest percentage of “I agree” answers was offered by students from Rijeka (87.6%) and Zagreb (81.6%), and the highest percentage of negative answers was offered by students from Ljubljana (37.5%).

Responses to the statement “Endodontic treatment on an avital tooth with symptoms must be performed at least 7 days before the start of oncology therapy.” are shown in Table 6. Most students from Split and only half of the students from Sarajevo answered “I agree”.

Even greater variance in results, both within each faculty and between faculties, was shown in the answers to the statement “If a single-visit endodontic procedure cannot be performed on a tooth, extraction of that tooth is required.”. Students from Rijeka agreed for the most part (62.5% of them), while students from Zagreb offered the most “I disagree” answers (44.8%).

To the statement “Dentists also recommend alcohol-based mouthwashes.”, most respondents from Rijeka (75.0%) and the least respondents from Sarajevo answered “I disagree” (33.3%).

To the question “Would you recommend removing fixed orthodontic appliances before starting oncological treatment?” (Table 7) the highest percentage of respondents from Sarajevo and the lowest percentage of respondents from Zagreb answered affirmatively. A relatively large percentage of “I do not know” answers was offered by students from all faculties, mostly students from Zagreb.

To the question “Do you think that it is best for the patient not to wear mobile prosthetic replacements during the oncology therapy?”, most respondents from Rijeka answered affirmatively (75.0%), and the most negative answers were offered by respondents from Zagreb (42.1%).

To the question “Can implants osseointegrate into the irradiated maxilla/mandible?”, the largest percentage of positive answers was offered by respondents from Rijeka (43.8%), while the largest percentage of negative answers was offered by respondents from Ljubljana (50.0%).

Most respondents from Rijeka (81.3%) answered affirmatively to the question “Do you think that supragingival and subgingival scaling should be delayed for some time after the end of oncology therapy?”, and the most negative answers were offered by respondents from Ljubljana (87.5%).

**Figure 1:**
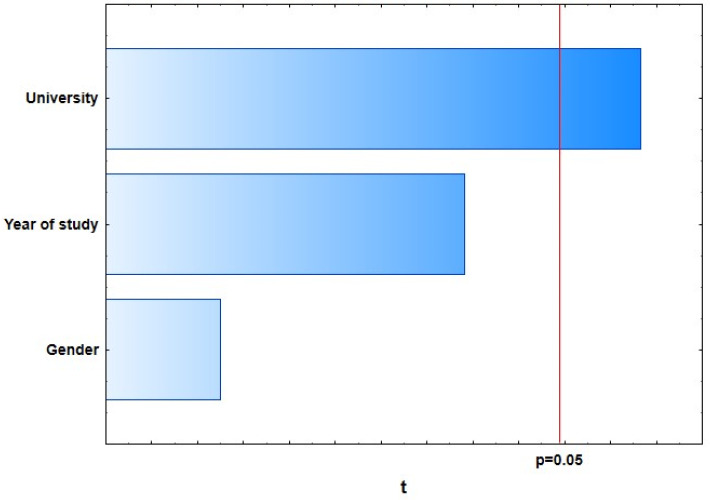
A higher incidence of caries in oncology patients has not been proven.

**Figure 2:**
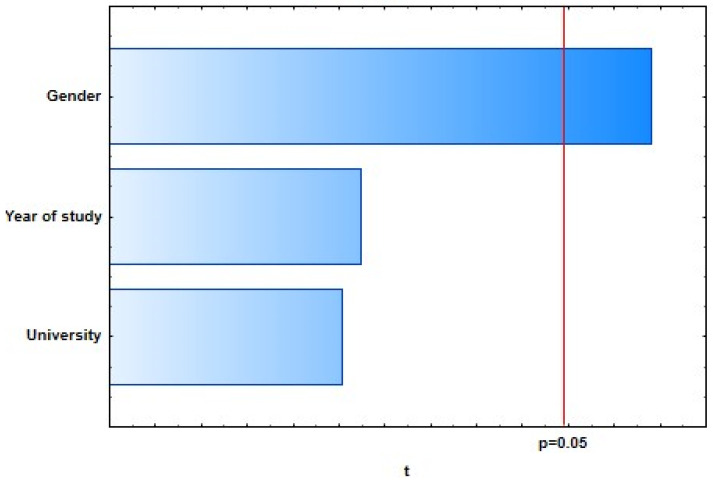
The ideal time to extract teeth with a poor prognosis is 3 weeks before starting oncology treatment.

**Figure 3:**
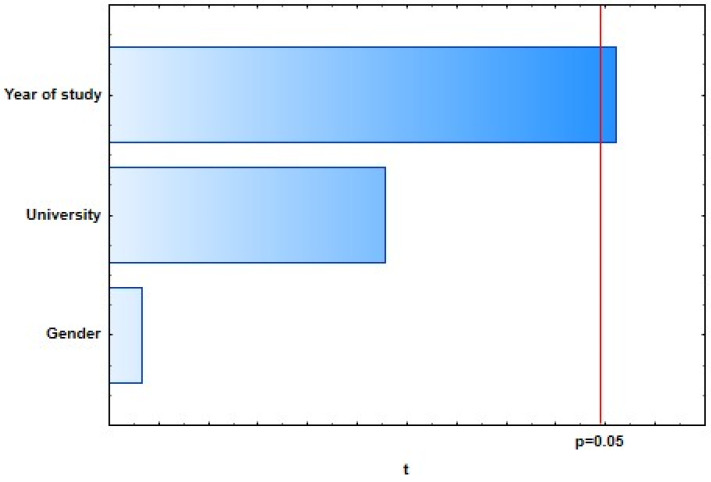
If a single-visit endodontic procedure cannot be performed on a tooth, extraction of that tooth is required.

**Figure 4:**
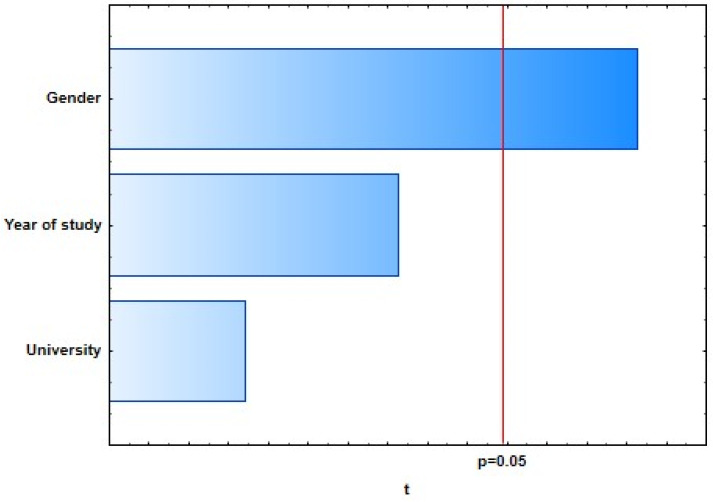
Dentists also recommend alcohol-based mouthwashes.

With the general regression model of 10 selected dependent variables representing students’ knowledge of dental care in oncology patients, four of them showed a weak, statistically significant correlation with predictor variables: “There is no proven higher incidence of caries in oncology patients.” (*R* = 0.25; *p* = 0.0373), “The ideal time for extraction of a tooth with a poor prognosis is 3 weeks before the start of oncology treatment.” (*R* = 0.25; *p* = 0.0376), “If a single-visit endodontic procedure cannot be performed on the tooth, extraction of that tooth is required.” (*R* = 0.20; *p* = 0.0462), and “Dentists also recommend alcohol-based mouthwashes.” (*R* = 0.24; *p* = 0.0419) (Figure 1, Figure 2, Figure 3 and Figure 4).

Although certain differences in the level of knowledge across faculties were observed, they were not statistically significant when applied in the Kruskal–Wallis ANOVA test.

## 4. Discussion

The main goal of this study was to determine whether the students of dental medicine from five different universities from three neighboring countries have enough knowledge about dental care of patients on oncology therapy. School programs are quite similar across all dental faculties. Respondents were mainly female students, and the majority of respondents were students in their fourth year of study. It seems that the younger students are more willing to respond to surveys or are more willing to test their knowledge. Older colleagues are either uninterested in completing surveys or overburdened with learning and have no desire to spend time completing surveys. We expected just the opposite, i.e., more responses from older years, as one form of testing their knowledge before graduation. According to the answers of 140 students from the last 3 years of studies, a conclusion is that they did not learn enough about this specific topic. Similar results were reported by Walton et al. in their study from 1992 when they examined the quality of dental students’ education in the field of oncology. Students’ knowledge about oncological patients was assessed on the basis of an exam results after a summer course at UCLA for 2 consecutive years of third-year dental students (1990 and 1991). In the same study, students were asked about risk factors for developing squamous cell carcinoma; 69% of students in 1990 and 77% in 1991 offered the correct answer [6]. This study showed similar results, whereby students showed good knowledge about the incidence of head and neck tumors cancer and the histological type of the cancer. They also knew that two-thirds of head and neck tumors occur in men. On questions about the prevention and treatment of complications of head and neck cancer treatment (ORN), 69% of students in 1990, and 77% in 1991 offered the correct answer [7].

The 2007 study conducted in Great Britain [8] investigated the differences in awareness of oral cancer between future doctors of medicine and dentistry. According to the answers on the 12-question questionnaire that included questions about an oral examination of patients, knowledge about risk factors and advising patients about them, the clinical appearance of oral precancerous lesions and oral cancer, and clinical care procedures of oncology patients, the authors concluded that respondents did not have enough knowledge [8]. In this research, to the question “Do you think you have enough knowledge to provide dental care for cancer patients?”, 45.7% of students from Split, Zagreb, Rijeka, Sarajevo, and Ljubljana answered that they think they are not educated enough. Nevertheless, there was a willingness for additional education on dental care for oncology patients.

In this study, the statement “Potentially long-lasting and most serious side-effect of radiotherapy is osteoradionecrosis (ORN)”, 81.4% of respondents answered correctly by choosing the answer “I agree with the statement”. Poorer knowledge was shown regarding radiation doses effects on ORN development and the potential antibiotic prophylaxis of ORN. To the claim “A total radiation dose greater than 40 Gy poses a risk to ORN.”, most of the answers offered were “I am not sure” (47.9%). To the question “Do you think that, before tooth extraction after oncology therapy, a patient is safe from ORN?”, only 55% of all respondents agreed that this was not true. According to these results, it is evident that knowledge about ORN as the most serious consequence of treatment is not satisfactory, and there is a need for additional education of students on this topic.

As for blood cell counts values and their importance for dental care of oncological patients, the research of Walton et al. [7] reported that approximately 64% of students showed a good knowledge of blood cell counts. Comparing results with this study, 73.6% of respondents answered that they agree to the statement “Before oral surgical treatment of a patient on chemotherapy, analysis of CBC and WBC counts is mandatory.”, and 64.3% of respondents answered affirmatively to the statement “In case an emergency invasive dental procedure must be performed, and the platelet count is less than 50000 cells/mm^3^, platelet transfusion is required.”, which is a similar finding to Walton’s study. Furthermore, to the statement “During chemotherapy, if the granulocyte count is less than 2000 cells/mm^3^, antibiotic prophylaxis is required.”, 60.7% of respondents answered affirmatively. It is evident that students are aware of the need to analyze CBC and WBC counts of patients.

A study by Alpöz et al. conducted in 2013 on Turkish final-year dental students examined students’ knowledge of oral complications that occur in oncology patients and their treatment, as well as the role of dentists in implementing necessary dental care protocols. Oral complications of chemotherapy and head and neck radiotherapy were accurately recognized by 87% of students [9]. This study obtained similar results; 84.3% of respondents answered correctly to the statement “Mucositis is an acute consequence of head and neck radiotherapy.”. The study by Singh et al. [10] reported that, due to radiation, some of the cells of oral mucosa die during or at the end of second week of therapy. Due to this fact, the mucous membrane will become red and inflamed. This clinical condition is known as mucositis. After the completion of radiotherapy, the mucosa begins to heal rapidly, and most healing is complete by about 2 months [11,12].

Radiation caries, a rampant form of dental decay, occurs in individuals who receive a course of radiotherapy that includes exposure of the salivary gland [10]. In a Turkish study on the question “Do dental caries progress faster after head and neck radiotherapy?”, 84.4% of students offered the correct answer. Our students also showed good knowledge by responding to the statement “There is no proven higher incidence of caries in oncology patients.”, whereby 77.9% disagreed with the statement. Moreover, 92.9% of students agreed with the statement “Pre-therapy assessment of an oncology patient must include a detailed clinical examination of the oral cavity and radiological imaging.”, thus showing a high level of knowledge about pre-therapy guidelines.

The guidelines presented by the Multinational Association of Supportive Care in Cancer (MASCC) and the International Society of Oral Oncology (ISOO) suggest the use of a soft toothbrush, dental floss with wax, and mouthwash several times after brushing your teeth [13]. Similarly, the guidelines of the British Society for Disability and Oral Health from 2018 recommend the use of a manual or electric brush with medium bristles, with the additional use of dental floss and interdental brushes. If brushing becomes too painful, the toothbrush can be replaced with a soft one, but they pointed out that soft toothbrushes are not as effective in controlling plaque [4]. Turkish students are not so familiar with pre-therapeutic oral patient evaluation, while students in this study were familiar with the instructions in oral hygiene and as many as 85% agreed with the statement “Patients undergoing head and neck radiotherapy should use soft brushes.”.

To the question about the ideal period for oral surgery before the start of oncological treatment, only 27.3% of Turkish students offered the correct answers. To the statement “The ideal time for extraction of a tooth with a poor prognosis is 3 weeks before the start of oncology treatment.”, 65.7% of students in this study answered affirmatively. The correct answer to the statement “Endodontic treatment on an avital tooth with symptoms must be done at least 7 days before the start of oncology therapy.” was offered by 59.3% of respondents. To the statement “If a single-visit endodontic procedure cannot be performed on a tooth, extraction of that tooth is required.”, 39.3% of students agreed.

In conclusion we can state that, although dental students learned about oncological therapy, their knowledge and readiness for providing dental care to oncological patients is still not enough. Most students are familiar with the incidence and most common type of head and neck tumors, while knowledge about tumor treatment and the side-effects of radiation therapy and/or chemotherapy is weak. Students did not show satisfactory knowledge about osteoradionecrosis, which is the most serious side-effect of radiotherapy; therefore, the emphasis on additional education should be the greatest in this area.

A good thing is that the surveyed students are aware of their lack of knowledge, and they want to learn more. Teaching staff should be aware of the situation and try to offer more information and practice in providing dental care for oncology patients. Perhaps the best way for that is to introduce courses to the curriculum that will prepare future dentists to work with oncology patients.

The limitations of this study were the small number of participants and the large difference in the number of responses per university. A similar study with a greater number of participants is needed to gain better insight into the dental students’ knowledge.

## Figures and Tables

**Table 1:** Frequency and percentage of respondents by gender for the five selected faculties of dental medicine.

	Ljubljana		Rijeka		Split		Sarajevo		Zagreb		In total	
	*N*	%	*N*	%	*N*	%	*N*	%	*N*	%	*N*	%
Female	5	62.5	14	87.5	38	79.2	23	76.7	35	92.1	115	82.1
Male	3	37.5	2	12.5	10	20.8	7	23.3	3	7.9	25	17.9
Total	8		16		48		30		2	38	140	

*N*—number of respondents, %—percentage of respondents.

**Table 2:** Frequency and percentage of respondents according to the year of study for five selected faculties of dental medicine.

Year of Study	Ljubljana		Rijeka		Split		Sarajevo		Zagreb		In total	
	*N*	%	*N*	%	*N*	%	*N*	%	*N*	%	*N*	%
**4**	4	50	7	43.8	16	33.3	17	56.7	20	45.7	64	45.8
**5**	0	0	9	56.2	16	33.3	13	43.3	7	32.1	45	32.1
**6**	4	50	0	0	16	33.3	0	0	11	22.1	31	22.1

*N*—number of respondents, %—percentage of respondents.

**Table 3:** Frequency and percentage of respondents with respect to responses to the statement “Total radiation dose greater than 40 Gy poses a risk to ORN.” for five selected faculties of dental studies.

	Ljubljana		Rijeka		Split		Sarajevo		Zagreb	
	*N*	%	*N*	%	*N*	%	*N*	%	*N*	%
I agree	5	62.5	8	50	17	35.4	15	50	17	44.7
I am not sure	2	25.0	8	50	23	57.5	15	50	19	50
I disagree	1	12.5	0	0	8	7.1	0	0	2	5.3

*N*—number of respondents, %—percentage of respondents.

**Table 4:** Frequency and percentage of respondents regarding the answers to the statement “In case an emergency invasive dental procedure must be performed, and the platelet count is less than 50,000 cells/mm^3^, platelet transfusion is required.” for five selected faculties of dental medicine.

	Ljubljana		Rijeka		Split		Sarajevo		Zagreb	
	*N*	%	*N*	%	*N*	%	*N*	%	*N*	%
I agree	5	62.5	8	50.1	24	50	24	**80**	30	76.4
I am not sure	2	25.0	5	31.3	19	39.6	5	16.7	7	18.4
I disagree	1	12.5	3	**18.8**	5	10.4	1	3.3	2	5.2

*N*—number of respondents, %—percentage of respondents.

**Table 5:** Frequency and percentage of respondents regarding the answers to the statement “During chemotherapy, if the granulocyte count is less than 2000 cells/mm^3^, antibiotic prophylaxis is required.” for five selected faculties of dental medicine.

	Ljubljana		Rijeka		Split		Sarajevo		Zagreb	
	*N*	%	*N*	%	*N*	%	*N*	%	*N*	%
I agree	2	25.0	12	**75.1**	27	**56.3**	20	**66.7**	24	**63.2**
I am not sure	3	37.5	2	12.5	19	39.6	10	33.3	12	31.6
I disagree	1	12.5	2	12.5	2	4.2			2	5.2

N—number of respondents, %—percentage of respondents.

**Table 6:** Frequency and percentage of respondents regarding the answers to the statement “Endodontic treatment on an avital tooth with symptoms must be performed at least 7 days before the start of oncology therapy.” for five selected faculties of dental medicine.

	Ljubljana		Rijeka		Split		Sarajevo		Zagreb	
	*N*	%	*N*	%	*N*	%	*N*	%	*N*	%
I agree	5	62.5	9	60	31	**64.6**	15	**50**	23	60.5
I am not sure	2	25.0	4	26.7	13	27.1	7	23.3	8	21.1
I disagree	1	12.5	2	13.3	4	8.4	8	26.7	7	18.5

N—number of respondents, %—percentage of respondents.

**Table 7:** Frequency and percentage of respondents regarding the answers to the question “Would you recommend removing fixed orthodontic appliances before starting oncological treatment?” for five selected faculties of dental medicine.

	Ljubljana		Rijeka		Split		Sarajevo		Zagreb	
	*N*	%	*N*	%	*N*	%	*N*	%	*N*	%
Yes	6	75.0	12	75.0	33	68.8	26	**86.7**	19	**50.0**
No					2	4.2	1	3.3	6	15.8
I do not know	2	25.0	4	25.0	13	27.1	3	10.0	13	**34.2**

*N*—number of respondents, %—percentage of respondents.
